# Lead level in seminal plasma may affect semen quality for men without occupational exposure to lead

**DOI:** 10.1186/1477-7827-10-91

**Published:** 2012-11-08

**Authors:** Hsien-Ming Wu, Dan-Tzu Lin-Tan, Mei-Li Wang, Hong-Yuan Huang, Chyi-Long Lee, Hsin-Shih Wang, Yung-Kuei Soong, Ja-Liang Lin

**Affiliations:** 1 Department of Obstetrics and Gynecology, Chang Gung Memorial Hospital, Chang Gung University School of Medicine, Taoyuan, Taiwan, ROC; 2 Division of Nephrology and Clinical Toxicology, Chang Gung Memorial Hospital, Chang Gung University School of Medicine, Taoyuan, Taiwan, ROC

**Keywords:** Lead, Infertility, Non-occupational exposure, Seminal plasma, Sperm count

## Abstract

**Background:**

Infertility affects approximately 10–15% of reproductive-age couples. Poor semen quality contributes to about 25% of infertile cases. Resulting from the direct effect on testicular function or hormonal alterations, heavy metals exposure has been related to impaired semen quality. The objective of this study was to assess the level of lead in the seminal plasma in men without occupational exposure to lead, and to determine the relationship between semen quality and lead concentration in the semen.

**Methods:**

This is a prospective and nonrandomized clinical study conducted in University infertility clinic and academic research laboratory. Three hundred and forty-one male partners of infertile couples undergoing infertility evaluation and management were recruited to the study. Semen samples collected for the analyses of semen quality were also used for the measurement of lead concentrations. Semen samples were evaluated according to the WHO standards.

**Results:**

All subjects were married and from infertile couples without occupational exposure to lead. There is a significant inverse correlation between the lead concentration in seminal plasma and sperm count. A higher semen lead concentration was correlated with lower sperm count, but not with semen volume, sperm motility or sperm morphology as assessed by simple linear regression.

**Conclusions:**

We found that semen lead concentration was significantly higher among the patients with lower sperm count. To our knowledge, this is the first study to demonstrate that a high level of lead accumulation in semen may reduce the sperm count contributing to infertility of men without occupational exposure to lead.

## Background

Infertility affects approximately 10–15% of reproductive-age couples. Poor semen quality contributes to about 25% of infertile cases [1]. The causes of poor semen quality is complex. An increasing number of reports suggest that the environmental, industrial and dietary agents may affect male fertility in human [2-5].

The evidences for decreasing quality of semen in men during the past 50 years are significant [6,7]. This semen quality decline has been suggested to be associated with exposure to the environmental, industrial and dietary toxins. An increase in the human population, rapid industrialization, and motorized vehicular traffic are believed to be responsible for the increased release of toxic metals into the environment. Heavy metals are possible pollutants that may be harmful to semen quality [8]. Lead exposure can cause adverse effects on both male and female reproductive systems. Lead in seminal plasma may be increased by high local environmental, industrial and dietary exposure. Resulting from the direct effect on testicular function or hormonal alterations, heavy metals exposure has been related to impaired semen quality [9,10]. Blood tests of heavy metals levels have been believed to the standard procedures for the exposure index in toxicological studies, but recent data suggest that it may be inadequate to reveal heavy metals accumulation in the male reproductive tract. Therefore, heavy metals concentrations in seminal plasma could provide a better evaluation of the effects of heavy metals exposure in reproduction [11,12].

An increasing exposure to the environmental, industrial and dietary toxins is believed to be related to poor semen quality. Our study's primary purpose is to determine whether semen concentration of lead in men without occupational exposure to lead is associated with semen quality and reproductive outcome.

## Methods

### Study populations

Three hundred and forty-one male partners of infertile couples attending the reproductive center of Lin-Kou medical center, Chang Gung Memorial Hospital and undergoing infertility evaluation were recruited to the study. A questionnaire survey collected data regarding patient occupation, smoking status, body height, body weight, marital status, infertility history and other patient data. Patients with a history of heavy metals exposure or who resided in areas known to have heavy metals contamination were excluded from this study. Semen samples collected for the analyses of semen quality were also used for the measurement of metal concentrations. All samples were collected by masturbation after 3 to 5 days of sexual abstinence. The medical charts of patients were reviewed. All data collection was approved by the Institutional Review Boards at Chang Gung Memorial Hospital (IRB number: 98-3916B).

### Semen analysis

Routine semen analysis was performed to assess sperm quality parameters, including semen quantity, sperm count, sperm motility, and sperm morphology according to the World Health Organization (WHO) method [13], after liquefaction at 37°C for 30 min and within 1 h of semen collection. The criteria for normozoospermia were a concentration of ≧ 15 × 10^6^/ml, with sperms of progressive motility more than 32% of spermatozoa, and normal morphology with oval-shaped head and no irregularities of tail in at least 30% of the spermatozoa.

### Lead analyses

Approximately 100-μl of seminal plasma was digested with 500-μl of super-grade 0.8 M HNO3 in a glass tube. The residue was dissolved in 1 ml of 1% HNO3 and applied to a graphite tube for detection of lead by atomic absorption spectrophotometer (Varian spectraAA 200Z, USA). The recovery of lead in spiked semen samples was 97%, respectively. The instrument was calibrated using 5, 10, and 15 μg/l standards for lead, respectively. A sample blank was prepared with each set of samples to control for possible metal contamination from external sources. The level of detection for lead was 0.1 μg/l, respectively. This study utilized both internal and external quality-control procedures, and obtained consistently satisfactory results. A certified commercially prepared product (Seronorm Trace Elements, Sero AS, Billingstads, Norway) was employed to check intra-batch accuracy and ensure inter-batch standardization. The coefficient of variation for lead measurement was ≤ 5.0%. External quality control was maintained via participation in two major programs: National Quality-Control Program conducted by the government, and the international program run by the College of American Pathologists.

### Statistical analysis

The Cox proportional-hazards model was employed to determine the significance of the variables for predicting the pregnancy during the study period. The differences between the two groups were analyzed with a Chi-square test combined with a Fisher test, and Student's *t*-test. The Mann–Whitney *U* test was employed for data that were not normally distributed. A simple and multiple linear regression analysis were performed to identify the relationship between seminal plasma lead concentrations and other variables. All P values were two-tailed, and all results are presented as means±SD. The P value less than 0.05 was considered statistically significant.

## Results

### Demographic characteristics

Three hundred and forty-one males comforming to the inclusion criteria were recruited in the study. Demographic characteristics for all subjects are presented in Table 1. The males ranged in age from 28 to 44 years with a mean age of 34.9 (S.D. = 3.7). The mean body mass index of the subjects was 22.6 (S.D.=1.6). The mean semen volume of the subjects was 3.0 ml, mean sperm concentration was 63.4 ×10^6^/ml and the average percentage of total progressively motile sperm was 60.1%. The average lead concentration was 2.19 μg/l as shown in Table 1. All subjects were married and infertile couples without occupational exposure to lead.

**Table 1:** The base-line characteristics of study patients (n=341)

**Variables**	**Mean± standard deviation (range) or number (%)**
Age (Y/O)	34.9±3.7 (28–44)
Body mass index(kg/m^2^)	22.6±1.6 (19.2-26.1)
Smoking	157 (46.0%)
Semen lead conc.(μg/l)	2.19±1.45 (0.08-9.50)
Semen volume (ml)	3.0±1.2 (0.3-8.0)
Sperm count (x10^6^)	63.4±49.6 (0.0-368.0)
Sperm motility (%)	60.1±20.2 (0.0-97.4)
Sperm morphology (%)	58.0±17.8(0.0-94.9)

### Lead concentration and semen quality

There is a significant inverse correlation between the lead concentration in seminal plasma and sperm count described in Table 2. A higher semen lead concentration was correlated with lower sperm count, but not with semen volume, sperm motility or sperm morphology as assessed by simple linear regression in Table 2.

**Table 2:** The relations between semen lead concentrations and semen volume, sperm count, sperm motility and sperm morphology assessed by simple linear regression (N=341)

**Variables**	**R values**	**P**
Age (Y/O)	−0.011	0.8443
Body mass index(kg/m^2^)	0.105	0.0529
Semen amount (ml)	0.037	0.4964
Sperm count (x10^6^)	0.130	0.0165
Sperm motility (%)	0.004	0.9448
Sperm morphology (%)	0.002	0.9755

### Sperm count and lead concentration

**Figure 1:**
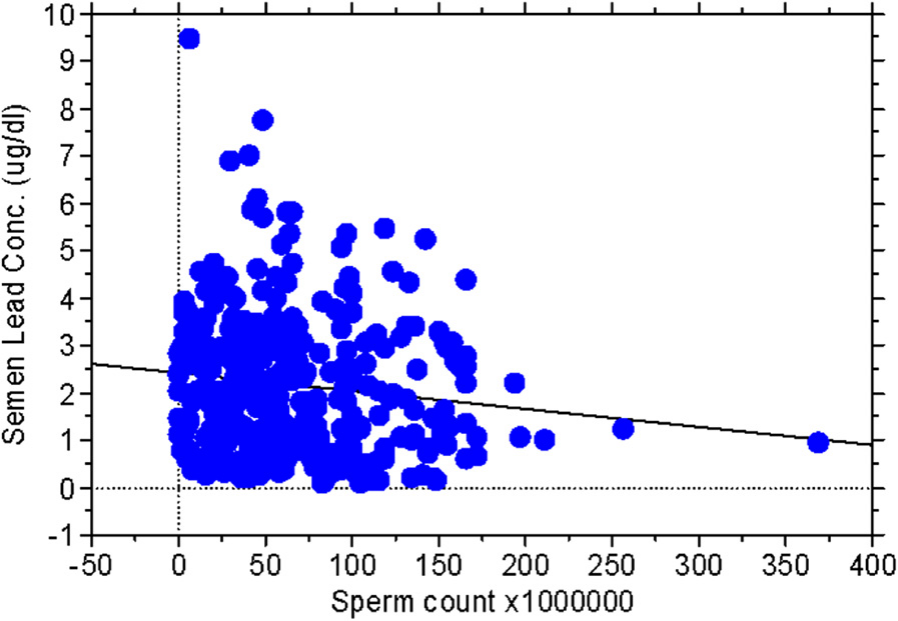
The relation between sperm count and semen lead concentration (assessed by simple linear regression analysis, r=0.130; p=0.0165; N=341).

After adjusting age, body-mass index and smoking by multiple linear regressions, Table 3 showed that sperm count did not correlate with age, body-mass index and smoking but with semen lead concentration (beta coefficients: -4.44±1.87, *P*=0.0181). After assessing by simple linear regression analysis, Figure 1 demonstrated that the sperm count was negatively associated with semen lead concentration (r=0.130, *P*=0.0165).

**Table 3:** The relations between sperm count and semen lead concentrations after adjusted age, body-mass index and smoking by multiple linear regressions (N=341)

**Variables**	**Beta coefficients± SE (standard error)**	**P**
Age (Y/O)	1.1±0.7	0.1433
Body mass index(kg/m^2^)	0.4±2.3	0.8514
Smoking	−0.2±5.6	0.9699
Semen lead	−4.44±1.87	0.0181

## Discussion

To obtain an accurate characterization of a male fertility status, semen analysis is a well established procedure. The basic semen analysis measures sperm concentration, sperm motility, and sperm morphology. To elucidate semen abnormalities, further evaluation is necessary to interpret the defect. Several environmental and endocrine factors may cause male infertility. The goal of the study is to evaluate the level of lead in the seminal plasma in male partners of infertile couples undergoing infertility evaluation, and to observe the association among sperm quality, pregnancy rate and lead concentration in the semen. The major finding in the study is that lead concentration in the seminal plasma contributes to the poor sperm count in the patients undergoing infertility evaluation and treatment. The patients in this study had no histories of lead poisoning or occupational exposure to lead. This indicates that chronic systemic low-level exposure to environmental toxicants such as lead may damage the reproductive function in general population. To our knowledge, this is the first study to show that low level of lead accumulation in seminal plasma may affect sperm amount for men without occupational exposure to lead. Exposure to environmental toxicants may result in poor-quality gametes and aneuploidy in gametes [14,15]. Some studies demonstrated that lead may lessen normal sperm metabolism through suppressing the sperm creatine kinase, contributing to infertility in men [16]. The relationship between such exposures and poor-quality gametes needs to be addressed in future study.

The previous studies demonstrated that the infertile couples without occupational exposure to lead exhibited high blood lead concentrations, attributable to poor IVF outcome related to poor oocyte quality and altered sperm function [17-19]. Some studies indicated that effects of smoking on blood lead levels in workers may affect semen quality [20]. However, the mechanisms of the actions underlying its toxic effects on male reproductive ability remain controversial. The gonadotropins bind specific receptors on the reproductive cell surface to stimulate gonadal steroidogenesis and gametegenesis. Since these receptors for gonadotropins are localized on the surface of the cell membrane, membrane integrity is important for gonadotropic binding, steroidogenesis, and gamete growth. The lead concentration in the tissue could affect hormone receptor kinetics, enzyme activities and hormone secretion [3]. Although environmental exposure to lead may impair spermatogenesis, as shown in several animal studies [3,21], similar data in human are generally limited. The above-mentioned data demonstrate that heavy metals intoxication alters steroidgenesis and gamete growth. Environmental low-level exposure to lead is a rising health issue, and more studies are encouraged to elucidate the lowest adverse effects of lead concentrations in biological fluids and tissue levels. The threshold level has been difficult to establish due to the selection of the exposure indicator and the reproductive endpoints [22]. Lead can be measured in reproductive organs, including prostate and seminal vesicles. Lead appears to accumulate in the epididymis, however, the distribution of lead in male reproductive tracts has not been well established. The association observed in our study between lead in semen compartments and sperm count is significant. The association between lead concentration in semen and sperm count may result from lead-detrimental spermatogenesis rather than lead-altered hypothalamic-pituitary-gonadal function [23]. Further studies are required to verify the impact of lead on semen environment and spermatogenesis.

Although blood lead level reflects recent exposure to lead, it is unclear whether the adverse health effects of lead that are observed are related to current or cumulative exposures. The previous studies indicate that workers exposed to lead had lower sperm count and higher teratozospermia count [24,25]. An negative correlation between blood lead concentrations and poor semen quality has been shown [17]. However, no association was observed between seminal plasma lead concentrations and semen quality in some studies [8,26]. The possible factors for the drawbacks are limited detecting methods and less sensitive examinations of environmental effects in seminal plasma lead concentrations. The results of the current study draw a more definite conclusion on the relationship between seminal plasma lead concentrations and sperm count in men without occupational exposure to lead.

## Conclusions

In summary, this is the first study to demonstrate that lead accumulation in semen may reduce the sperm count contributing to infertility of men without occupational exposure to lead. Our results suggest that efforts to reduce potential sources of environmental lead exposure may improve the fertile ability in infertility couples. Effects of potential reproductive toxicants such as lead on reproductive ability is difficult to determine due to varying lead sensitivity, duration of exposure, and tissue concentration. Further studies are required to clarify the complicated mechanisms of environmental exposure to lead and male infertility in general population.

## Competing interests

The authors declare that they have no competing interests.

## Authors' contributions

HMW, HYH, CLL, HSW, YKS and JLL participated in the design of the study and in developing the research; HMW, DTLT, MLW, HYH, CLL, HSW, YKS and JLL conducted the study at the outpatient clinic, and at the laboratory. All the authors contributed equally in writing the manuscript and in reviewing and revising it. All authors read and approved the final manuscript.

## References

[B1] Templeton A Infertility-epidemiology, aetiology and effective management Health Bull (Edinb) 1995 53 294 298 7490200

[B2] Eskenazi B Wyrobek AJ Sloter E Kidd SA Moore L Young S Moore D The association of age and semen quality in healthy men Hum Reprod 2003 18 447 454 10.1093/humrep/deg107 12571189

[B3] Benoff S Jacob A Hurley IR Male infertility and environmental exposure to lead and cadmium Hum Reprod Update 2000 6 107 121 10.1093/humupd/6.2.107 10782569

[B4] Auger J Eustache F Andersen AG Irvine DS Jorgensen N Skakkebaek NE Suominen J Toppari J Vierula M Jouannet P Sperm morphological defects related to environment, lifestyle and medical history of 1001 male partners of pregnant women from four European cities Hum Reprod 2001 16 2710 2717 10.1093/humrep/16.12.2710 11726600

[B5] Said TM Ranga G Agarwal A Relationship between semen quality and tobacco chewing in men undergoing infertility evaluation Fertil Steril 2005 84 649 653 10.1016/j.fertnstert.2005.03.052 16169398

[B6] Carlsen E Giwercman A Keiding N Skakkebaek NE Evidence for decreasing quality of semen during past 50 years Bmj 1992 305 609 613 10.1136/bmj.305.6854.609 1393072 PMC1883354

[B7] Shine R Peek J Birdsall M Declining sperm quality in New Zealand over 20 years N Z Med J 2008 121 50 56 19098968

[B8] Hovatta O Venalainen ER Kuusimaki L Heikkila J Hirvi T Reima I Aluminium, lead and cadmium concentrations in seminal plasma and spermatozoa, and semen quality in Finnish men Hum Reprod 1998 13 115 119 10.1093/humrep/13.1.115 9512240

[B9] Alexander BH Checkoway H van Netten C Muller CH Ewers TG Kaufman JD Mueller BA Vaughan TL Faustman EM Semen quality of men employed at a lead smelter Occup Environ Med 1996 53 411 416 10.1136/oem.53.6.411 8758037 PMC1128498

[B10] Telisman S Colak B Pizent A Jurasovic J Cvitkovic P Reproductive toxicity of low-level lead exposure in men Environ Res 2007 105 256 266 10.1016/j.envres.2007.05.011 17632096

[B11] Apostoli P Kiss P Porru S Bonde JP Vanhoorne M Male reproductive toxicity of lead in animals and humans. ASCLEPIOS Study Group Occup Environ Med 1998 55 364 374 10.1136/oem.55.6.364 9764095 PMC1757597

[B12] Plechaty MM Noll B Sunderman FW Jr Lead concentrations in semen of healthy men without occupational exposure to lead Ann Clin Lab Sci 1977 7 515 518 931355

[B13] Organization WH WHO laboratory manual for the Examination and processing of human semen 2010 5 WHO Press, Switzerland

[B14] Morita Y Perez GI Paris F Miranda SR Ehleiter D Haimovitz-Friedman A Fuks Z Xie Z Reed JC Schuchman EH Oocyte apoptosis is suppressed by disruption of the acid sphingomyelinase gene or by sphingosine-1-phosphate therapy Nat Med 2000 6 1109 1114 10.1038/80442 11017141

[B15] Meirow D Epstein M Lewis H Nugent D Gosden RG Administration of cyclophosphamide at different stages of follicular maturation in mice: effects on reproductive performance and fetal malformations Hum Reprod 2001 16 632 637 10.1093/humrep/16.4.632 11278209

[B16] Ghaffari MA Motlagh B In vitro effect of lead, silver, tin, mercury, indium and bismuth on human sperm creatine kinase activity: a presumable mechanism for men infertility Iran Biomed J 2011 15 38 43 21725498 PMC3639739

[B17] Winder C Lead, reproduction and development Neurotoxicology 1993 14 303 317 8247405

[B18] Benoff S Cooper GW Paine T Hurley IR Napolitano B Jacob A Scholl GM Hershlag A Numerical dose-compensated in vitro fertilization inseminations yield high fertilization and pregnancy rates Fertil Steril 1999 71 1019 1028 10.1016/S0015-0282(99)00136-3 10360904

[B19] Kunzle R Mueller MD Hanggi W Birkhauser MH Drescher H Bersinger NA Semen quality of male smokers and nonsmokers in infertile couples Fertil Steril 2003 79 287 291 10.1016/S0015-0282(02)04664-2 12568836

[B20] Hsu PC Chang HY Guo YL Liu YC Shih TS Effect of smoking on blood lead levels in workers and role of reactive oxygen species in lead-induced sperm chromatin DNA damage Fertil Steril 2009 91 1096 1103 10.1016/j.fertnstert.2008.01.005 18342860

[B21] Hauser R The environment and male fertility: recent research on emerging chemicals and semen quality Semin Reprod Med 2006 24 156 167 10.1055/s-2006-944422 16804814

[B22] Wu HM Lin-Tan DT Wang ML Huang HY Wang HS Soong YK Lin JL Cadmium level in seminal plasma may affect the pregnancy rate for patients undergoing infertility evaluation and treatment Reprod Toxicol 2008 25 481 484 10.1016/j.reprotox.2008.04.005 18554863

[B23] Allouche L Hamadouche M Touabti A Chronic effects of low lead levels on sperm quality, gonadotropins and testosterone in albino rats Exp Toxicol Pathol 2009 61 503 510 10.1016/j.etp.2008.12.003 19188052

[B24] Robins TG Bornman MS Ehrlich RI Cantrell AC Pienaar E Vallabh J Miller S Semen quality and fertility of men employed in a South African lead acid battery plant Am J Ind Med 1997 32 369 376 10.1002/(SICI)1097-0274(199710)32:4<369::AID-AJIM8>3.0.CO;2-P 9258391

[B25] Lerda D Study of sperm characteristics in persons occupationally exposed to lead Am J Ind Med 1992 22 567 571 10.1002/ajim.4700220411 1442789

[B26] Aribarg A Sukcharoen N Effects of occupational lead exposure on spermatogenesis J Med Assoc Thai 1996 79 91 97 8868019

